# Community-based rehabilitation implementation for people with disabilities in South Africa: a protocol for a scoping review

**DOI:** 10.1186/s13643-021-01839-7

**Published:** 2021-10-28

**Authors:** Sithembiso Blose, Saul Cobbing, Verusia Chetty

**Affiliations:** 1 Discipline of Physiotherapy, School of Health Sciences, University of KwaZulu-Natal, Private Bag X54001, Durban, 4000 South Africa; 2 School of Health Sciences, University of KwaZulu-Natal, Westville Campus, Durban, South Africa

**Keywords:** Community-based rehabilitation, People with disabilities, South Africa

## Abstract

**Background:**

People with disabilities (PWDs) remain among the poorest and least empowered population. They experience limited access to basic services, especially in low- and middle-income countries (LMIC). The infringement of their human rights remains at an alarming level, despite the availability of the community-based rehabilitation (CBR) strategy and the United Nations Convention on the Rights of People with Disabilities (UNCRPD). CBR, as a strategy for poverty alleviation, social inclusion and equalisation of opportunity, has broadened its scope from a mere strategy for access to health and rehabilitation services to include education, livelihood, social inclusivity and empowerment. CBR is implemented across the world in the majority of LMIC signatories to the UNCRPD. South Africa is among the countries that are implementing CBR. However, the extent and the nature of implementation is not known. This study, therefore, aims to map out the empirical evidence of the implementation of CBR in South Africa.

**Method:**

The study is a scoping review based on the Preferred Reporting Items for Systematic Reviews and Meta-Analyses extended for Scoping Review (PRISMA-ScR) methodology. The information will be extracted and captured on a data charting template that will be used through each phase of the study. The review will be guided by the following research question validated by the amended population-concept-context framework according to the Joanna Briggs Institute methodology for scoping reviews: ‘An investigation into CBR implementation in South Africa.’ The search will be conducted in the following electronic databases Google Scholar, PubMed, Medline, and Cochrane, etc, using Boolean logic. Restrictions will be set for years (Jan. 2009–Dec. 2019), English language peer-reviewed studies based on South Africa. The search output will be screened for primary studies on Community based rehabilitation in South Africa. Two independent reviewers will conduct title and abstract screening to identify potential eligible studies. After which full-text screening on the potential eligible studies and assessed for inclusion by the two independent reviewers. The Mixed Method Appraisal Tool will be applied to assess the quality of the studies included in the review.

**Discussion:**

The gathered evidence from the selected studies will be discussed in relation to the research questions using a narrative to identify and explore emergent themes. The review will provide a baseline of evidence on the implementation of CBR and will highlight gaps regarding the implementation of CBR in a South African Context. The gaps identified will be used to develop a framework that will guide implementation of CBR in South Africa.

## Background

**Fig. 1:**
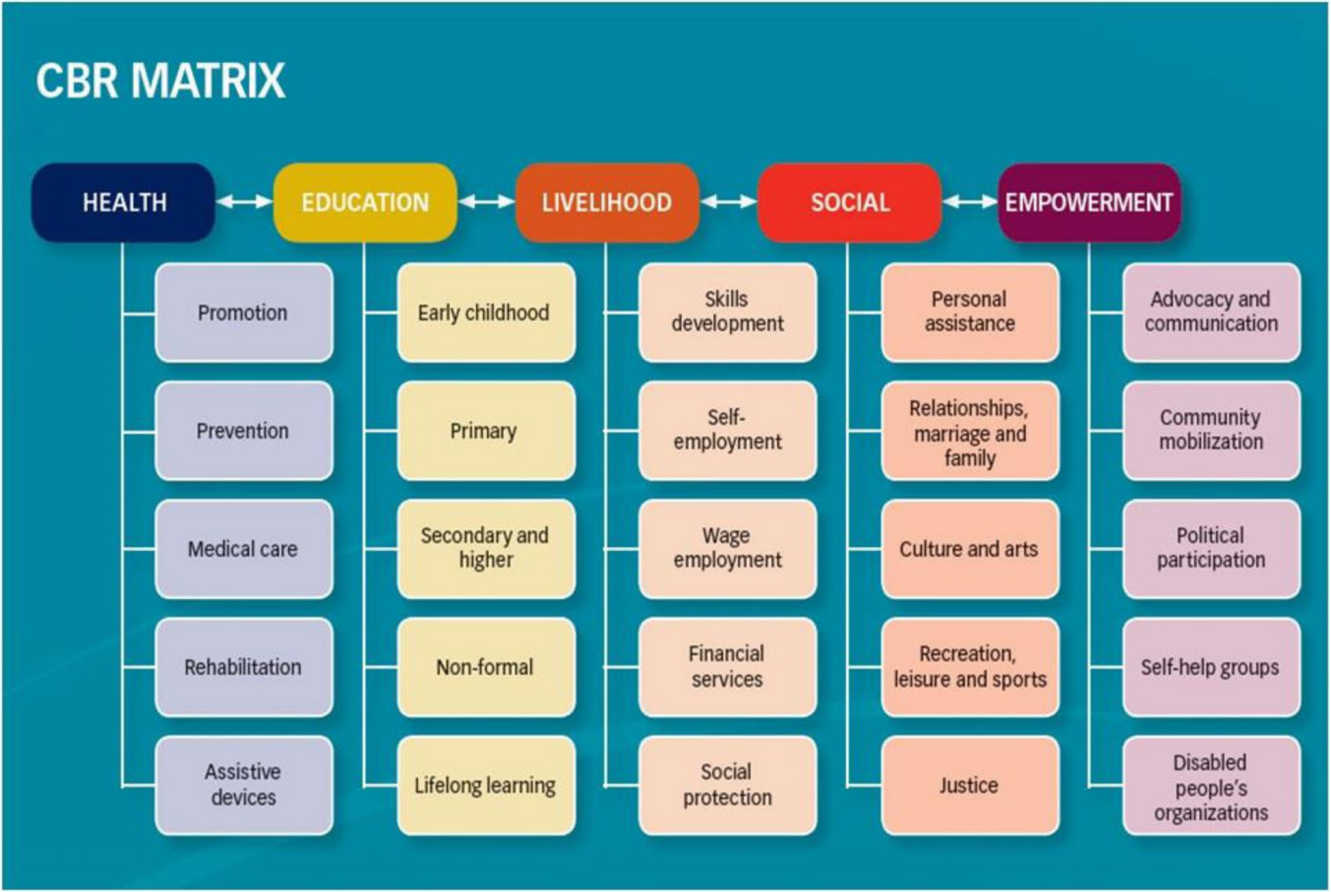
CBR matrix [1]

People with disabilities (PWDs) are among the poorest and least-empowered community members, especially in low- and middle-income countries such as South Africa (LMIC) [1, 2]. According to WHO estimates, 1 billion (15%) of the world’s population is made up of PWDs and 80% are PWDs from low- and middle-income countries [1]. South Africa has a prevalence of 7.5% of PWDs (Table 1), with Free State, Northern Cape and North West Provinces having the highest prevalence respectively. Gauteng and Western Cape Provinces have the least number of PWDs, respectively [3]. PWDs in South Africa still experience challenges in accessing basic human rights services which include healthcare (including rehabilitation), education, employment, and social inclusion [4].. These challenges persist despite the availability of the community-based rehabilitation (CBR) strategy which was developed by the World Health Organization in 1979 following the Alma Ata declaration [2, 5]. CBR is a strategy that is aimed at equalisation of opportunities, improving access to services, poverty alleviation and social integration of PWDs. The initial strategy was focused on access to health and rehabilitation services [6]. The scope of CBR has changed since the 2006 United Nations Convention on the Rights of People with Disabilities (UNCRPD), to a matrix (Fig. 1) that includes education, livelihood, social integration and empowerment [7]. Despite all these changes, PWDs remain in a poverty cycle and experience a gross infringement of their human rights, especially in LMICs [8, 9].

**Table 1:** Disability statistics – South Africa

Province	PWDs – *N* %
Western Cape	5.4
Eastern Cape	9.6
Northern Cape	11.0
Free State	11.1
KwaZulu-Natal	8.4
North West	10.0
Gauteng	5.3
Mpumalanga	7.0
Limpopo	6.9
**South Africa**	**7.5**

Africa’s endorsement of UNCRPD, following signing and ratification by over 40 countries, further strengthened the CBR African Network (CAN), which was founded in 2001 following the first CBR African region conference (https://afri-can.org, CBR African Network, 2019). CAN, as a non-profit organisation (NPO), aims at creating a platform for information sharing and encouraging research on CBR in Africa. South Africa is among the countries that have ratified the UNCRPD and has sought to align it to its constitution, legislative framework and policies in the post-apartheid era (https://www.gov.za/documents/white-paper-rights-persons-disabilities-official-publication-and-gazetting-white-paper).

The National Rehabilitation Policy (2000) of the Department of Health outlines its strategy for the policy implementation by including the re-orientation of service providers on CBR principles and outlining processes for developing rehabilitation services [10, 11]. Further, the Department of Health has developed a Framework and Strategy for Disability and Rehabilitation in South Africa (FSDR: 2015-2020), based on CBR philosophy, with the aim of improving service delivery from the community to tertiary level [12]. However, PWDs are still faced with numerous challenges, including a widening gap of unequal access to, and utilisation of, health services and rehabilitation [13].

The purpose of this study is to map out evidence of CBR implementation in South Africa. The study will seek to identify the stakeholders involved in the implementation of CBR and identify barriers and facilitators of CBR in South Africa. The study will also seek to understand the structure of CBR in South Africa and its nine provinces.

## Methodology

### Objective

To find the empirical evidence of CBR implementation in South Africa

### Identifying the research question

To what extent is CBR implemented in South Africa?

### Eligibility of research question

The study will follow the population concept context (PCC) framework to determine the eligibility of the research question. Refer to Table 2 below:

**Table 2:** Framework for determining eligibility of research question

P – Population	‘People with disabilities’ refers to all people that have long-term physical, mental, intellectual or sensory impairments, which in interaction with various barriers may hinder their full and effective participation in society on an equal basis with others. ‘Community health worker’ refers to a member of the community in which she/he works and who serves and responds to the health needs of the community. ‘Community rehabilitation worker’ is a member of the community that has undergone training on CBR and disability.
C – Concept	Community-based-rehabilitation
C – Context	South Africa

### Identification of relevant studies

Primary research articles published in peer-reviewed journals will form part of this study. Grey literature will also be included in the study. Different electronic databases will be used. These will include PubMed, Google Scholar, Cochrane database for Systemic Reviews and EBSCOhost of the University of KwaZulu-Natal library. Appropriate search terms will be identified and used. These terms include: ‘community-based rehabilitation’; ‘people with disabilities’; ‘community health worker’; ‘community rehabilitation worker’; Boolean terms, ‘and’, ‘or’ ‘not’will be used to separate keywords. A feasibility study using keywords was conducted as a pilot search. Once a search strategy has been developed, it will be adapted for each database. Each search will be documented to show details such as keywords, search engine, number of publications found/retrieved and date of search. Table 3 below is an example of a pilot search.

**Table 3:** Result of pilot research

Keywords searched	Search engine	Number articles found/retrieved	Date of search
((((((People with Disabilities)) OR (Disabled people)) AND (Community based rehabilitation))) AND (Community health worker)) OR (Community rehabilitation worker)	PubMed	482	11/11/2019
((((((People with Disabilities)) OR (Disabled people)) AND (Community based rehabilitation))) AND (Community health worker)) OR (Community rehabilitation worker)	EBSCOhost	220	11/11/2019

### Study selection

#### Eligibility criteria

The inclusion and exclusion criteria of the study will be led by the research question, in order to achieve accurate detection and selection of appropriate studies.

#### Inclusion criteria

The studies that include the following criteria will be included:

- Article published between 2009 and 2019 (primary studies)
- Studies that report on CBR in South Africa or southern Africa
- Studies that include CBR and mental health

#### Exclusion criteria

The studies that contain the following criteria will be excluded:

- Articles from countries outside of Africa, including those recognized as LMIC
- Commentaries on CBR implementation

### Charting of data

The information will be extracted and captured on a data charting template that will be used through each phase of the study, independently. The template, or chart, will be updated regularly as the process of scoping unfolds. This will allow for capturing all relevant information related to the research question. Appendix 1 is an example of the data chart.

### Collating, summarising and reporting results

The objective of the study is to identify evidence of implementation of CBR in South Africa. This research will seek to find existing information on the empirical evidence for CBR in South Africa. A thematic analysis will be thoroughly conducted on all full-text studies.

A qualitative analysis will be used to analyse the themes in relation to the research question as follows (but not limited to): CBR impact on people with disabilities; role of stakeholders in CBR implementation; barriers and facilitators of CBR implementation; and successes of CBR programmes. The Preferred Reporting Items for Systematic Reviews and Meta-Analysis (PRISMA) will be used as an approach to present the results of this study [14].

### Quality appraisal

An assessment of the quality of studies included will be done through quality appraisal steps using the mixed method appraisal tool (MMAT) version 2018 [15]. The MMAT is a critical appraisal tool that is designed and used for the appraisal stage of a systematic mixed studies review. The MMAT allows for the appraisal of the most common types of study design and methodology. The tool allows for examination of the quality of studies included, by assessing the aims, methodologies, study designs, data collection, presentation of findings, discussions and conclusions [16]. The evaluation and critical appraisal of grey literature will be done through the use of the Accuracy Authority Coverage Objectivity Date Significance (AACODS) checklist (Landford T. UC Library Guides; 2019)

## Discussion

The proposed scoping review study aims to map existing peer-reviewed and grey literature for evidence on implementation of CBR in South Africa. South Africa, as a developing country, is developing and introducing reforms in the health system, which will directly impact on people with disabilities. The mapping of literature to outline current trends and practices on CBR will provide empirical evidence that will assist in planning of services. The results of the study will also assist in identifying and outlining key stakeholders and their roles for CBR implementation. Barriers and facilitators identified will be used to further develop services and enhance the programmes that are aimed for people with disabilities. Any limitations of the scoping review will also be discussed in this section.

## Conclusion

The findings of the review will be useful in assessing the situation in South Africa regarding the rendering services to people with disabilities through the CBR strategy. The engagement of various stakeholders and their roles in CBR implementation is key for the strategy. Therefore, the available literature will assist in establishing the extent to which South Africa is implementing CBR, while also highlighting the challenges and/or gaps.

## Data Availability

Not applicable.

## Appendix 1

### Data charting

**Table:**

Item	Finding
Year of publication	
Country of origin (where study conducted)	
Author/s	
Aims/purpose	
Study population	
Sample size (if applicable)	
Methodology	
Programme (initiation/evaluation/impact study)	
Outcomes	
Key findings related to research question/s	
Conclusion	

## Abbreviations

AACODS: Accuracy Authority Coverage Objectivity Data Significance
CAN: CBR African Network
CBR: Community-based rehabilitation
FSDR: Framework and strategy for disability and rehabilitation in South Africa
LMIC: Low- and middle-income countries
MMAT: Mixed method appraisal tool
NPO: Non-profit organisation
PRISMA: Preferred Reporting Items for Systematic Reviews and Meta-Analysis
PWD: People with disabilities
PCC: Population, concept, context
UNCRPD: United Nations Convention on the Rights of People with Disabilities
WHO: World Health Organization
